# Psychometric testing of the Swedish version of the measure of moral distress for healthcare professionals (MMD-HP)

**DOI:** 10.1186/s12910-023-00916-x

**Published:** 2023-05-30

**Authors:** Catarina Fischer-Grönlund, Margareta Brännström, Ulf Isaksson

**Affiliations:** 1 Department of Nursing, Umeå University, Linnaeus v 9, 90736 Umeå, Sweden; 2 Department of Nursing, Umeå University, Campus Skellefteå, 93187 Skellefteå, Sweden

**Keywords:** Moral distress, Instrument, Measure, Healthcare professionals, Psychometrics, Validation

## Abstract

**Background:**

Moral distress has been described as moral constraints and uncertainty connected with guilty feelings of being unable to give care in accordance with one’s values for good care. Various instruments to measure moral distress have been developed. The instrument measure of moral distress for healthcare professionals (MMD-HP) was developed to capture the experience and frequency of moral distress among various healthcare professionals. The MMD-HP has been translated and culturally adapted into the Swedish language and context; however, the translation has not been validated. Therefore, this study aimed to evaluate the validity and reliability of the Swedish version of the measure of moral distress for healthcare professionals (MMD-HP).

**Methods:**

Eighty-nine staff from various professions at a hospital in northern Sweden participated in the study. A confirmatory factor analysis was performed to check for consistency with the original version of the MMD-HP. To evaluate internal consistency, Cronbach’s alpha was calculated for each domain and for the scale as a whole.

**Results:**

The scale as a whole showed a Cronbach’s alpha of 0.96, with a range between 0.84 and 0.90 between the different subscales. A confirmatory factor analysis based on the original four-factor structure showed good fit indices with a χ^2^/df of 0.67, CFI at 1.00, TLI at 1.02 and NFI at 0.97. RMSEA was at 0.00, and SRMR was at 0.08. A comparison of the total score between three equally large groups of years of experience at the present workplace showed no significant differences (F = 0.09, df = 2, *p* = 0.912).

**Conclusions:**

We found that the Swedish version of the MMD-HP has shown validity and reliability for use in a Swedish context for measuring moral distress among health personnel.

## Background

The experience of moral distress has been described as a phenomenon experienced by healthcare professionals from various professional groups, such as registered nurses (RNs) [1], physicians [2], occupational therapists [3] and hospital social workers [4]. Moral distress was initially described by Jameton as feelings of frustration, anger and guilt caused by institutional obstacles to giving care in accordance with personal values and judgements [5]. According to Kälvemark [6], moral distress occurs when there are dissonances between organisational values and health care professionals’ value systems, such as being prevented from giving sufficient care due to institutional constraints [6]. Root causes of moral distress was described, such as having to give compromised patient care, compromised integrity within the team and damaged interactions with patients and their families [7, 8].

According to Lützen and Kvist moral distress is an emotionally draining condition [9]. In a study by Morley et al., moral distress was described as moral constraints, tension and uncertainty expressed by feelings of guilt, anger and frustration [10]. Hamric suggests that moral distress is closely connected to a person’s value system [11] and might be triggered by increased awareness of ethics in clinical practice [9]. Being prevented from acting in accordance with values of what constitutes good care may lead to compromised core values and compromised personal, professional, and moral integrity [11–13], leading to the risk for burnout [14] and leaving work [15].

A variety of reasons have been found to increase the level of experienced moral distress, such as general workplace distress [16], organisational issues [17], constraints and low staffing [18], insufficient teamwork [17] and poor communication within the team [8]. Studies show that a positive ethical climate [19] and work independence reduce the frequency of moral distress [20].

According to Gallagher, there is a crucial interrelationship between aspects of culture, organisation, self-scrutiny, collegial dialogue and moral distress [21]. Nurses have described seeking support among colleagues as a way to cope with moral distress [22]. Hamric states that healthcare professionals may be unaware of ethically challenging situations that lead to moral distress, and interventions may work to bring clarity to the situations and identify root causes [23]. Various interventions such as education [24] or moral empowerment programmes [25] have been found to positively reduce moral distress.

Various instruments to measure moral distress have been developed, such as Moral Distress Intensity, Ethical Distress [26], Moral Distress in Dementia Care Survey (MDDCS) [27] and the Moral Distress Thermometer [28]. Corley originally developed the Moral Distress Scale [29], which Hamric and Blackhall later shortened [15] and further revised to become the Moral Distress Scale-Revised (MDS-R) [7]. MDS-R is a 21-item questionnaire with six versions constructed to apply to various healthcare professions [7]. The instrument has been translated and validated in several different languages worldwide, for instance, Japanese [18], Farsi [30], Turkish [31] and Swedish [32]. In addition, the MDS-R has been used to measure moral distress in a variety of healthcare contexts, such as community care [33], intensive care [34], paediatric care [35] and psychiatric care [18]. The measure of moral distress for healthcare professionals (MMD-HP) was developed by Epstein et al. to capture the experience and underlying reasons for moral distress among various healthcare professionals. The instrument comprises 27 items and has a factor structure at a system level, patient level, team level related to patients and team level related to colleagues [36].

The MMD-HP has been translated and validated in a Japanese version [37] and in Spanish (MMD-HP- SPA)[38]. Total score of MMD-HP showed to have a good reliability (α = 0.93) [36], the Japanese version (α = 0.91) [37], MMD-HP- SPA (α = 0.97) [38].

This study is a part of a larger project (Ethics Com Study) with the purpose of evaluating the effect of interprofessional ethics communication in groups among healthcare professionals. One factor in the Ethics Com Study was to measure the effect of experienced moral distress. Although the Swedish version of the Moral Distress Scale-Revised questionnaire was available [32], the MMD-HP was considered the most suitable, since the instrument was developed to measure moral distress among healthcare professionals from various professions and contexts. The MMD-HP was translated and culturally adapted into the Swedish language and context [39]. A prerequisite for establishing the validity of the Swedish version of MMD-HP was to carry through psychometric testing.

This study aimed to evaluate the validity and reliability of the Swedish version of the measure of moral distress for healthcare professionals (MMD-HP).

## Methods

### Questionnaire

The Measure of moral distress-health care professionals (MMD-HP) was recently developed and validated [36], translated and culturally adapted into the Swedish context [39]. The questionnaire comprises 27 items with statements of ethically difficult situations or dilemmas in healthcare. Each item is scored according to the frequency (how often) and intensity (how distressing) of experienced moral distress. The scoring is on a five-point Likert scale, whereby frequency is 0 = never and 4 = very frequently, and Intensity is 0 = none and 4 = very distressing. Finally, the MMD-HP has two questions comprising whether a person has left or intends to leave their position due to moral distress. A composite score for each item is calculated by multiplying each item’s frequency and intensity level (0–16). A total score is calculated by summarising overall composite scores, ranging from 0 to 432, where high scores indicate a higher level of perceived moral distress [36]. An explorative factor analysis of the MMD-HP showed a four-factor structure which aims to capture the root causes of moral distress experienced by healthcare professionals. Factor 1 comprises system-level root causes, and factor 2 clinical root causes at the patient level. Factors 3 and 4 involve team-level root causes with a differentiation between these. Factor 3 involves compromises to integrity occurring within a team that can be perceived as a personal threat by a team member. In contrast, factor 4 concerns breakdowns in the team’s interactions with patients and families [36]. A confirmatory analyses [37] confirmed this four-factor structure.

The MMD-HP was first translated and culturally adapted into Swedish using the WHO guidelines 2020 [40]. The translation process was performed in four steps: forward translation from English to Swedish, backward translation from Swedish into English and cognitive interviews with an expert panel for validation [39]. Finally, in a fourth step, the Swedish version was pretested in face-to-face cognitive interviews with healthcare professionals from various professions and contexts [39]. In addition, data concerning participant characteristics, i.e. gender, occupation, etcetera, was collected.

### Study setting and participants for validation

This study is part of an intervention study to evaluate the effects of clinical ethics support (CES) among healthcare professionals (The Ethics Com Study: ClinicalTrials.gov Identifier: NCT05146102). Nine departments at a hospital in northern Sweden participated in the study, and the heads of the departments gave their consent to participate. All healthcare professionals working in the included departments were informed orally and in writing and asked to participate in the study. Staff (n = 89) from various professions accepted participation. The departments were specialised in emergency care, critical care, infection care, thoracic care, palliative care, neurorehabilitation and geriatric care. Participants are described in Table 1.

**Table 1:** Characteristics of the study sample

	N (%)	Med (IQR)
Women	68 (85.0)	
Age		
− 20	1.0(1.3)	
21–30	16 (20.0)	
31–40	14 (17.5)	
41–50	17 (21.3)	
51–60	24 (30.0)	
61–	8.0 (10.0)	
Year at present workplace		6 (2–12.3)
Year within the profession		14 (5–24.5)
Profession		
Enrolled nurse	24 (29.1)	
Registered nurse	34 (43.0)	
Physician	7.0 (8.9)	
Physio/ occupational therapists	15.0 (19.0)	

### Data collection

The questionnaire and an information letter were distributed to the participants by the ethical representative or the head of the department. The questionnaires were placed in a sealed envelope, returned to the ethical representative or head of the department and further collected by the researcher (CFG). In addition, a reminder was communicated to the ethical representative or head of the department by email or telephone twice during the period.

### Statistical analysis

Missing values were imputed with the median value for the whole sample on respective items. However, if a respondent had more than 10% missing values, the respondent was excluded from the analysis. This meant that the number of respondents in the analysis dropped from 89 to 80. The analysis began with an assessment of distributional properties – that is, distribution percentage and the number of missing values on item level. A correlation was done to check for relationships between the different domains. Floor and ceiling effects were analysed and considered present if > 15% of participants reported the lowest or the highest response option of an item [42].

Since this was a validation of a Swedish version of an instrument that was a priori defined, a confirmatory factor analysis was performed to check for consistency with the original version. For assessing goodness of fit, chi-square (χ^2^), chi-square/degrees of freedom (χ2/df), Comparative Fit Index (CFI), Tucker-Lewis Index (TLI), root mean square error of approximation (RMSEA) and standardised root mean squared residual (SRMR) were used. The diagonally weighted least squares (DWLS) estimator was used, since this is designed for use with ordinal data and assumes a normal latent distribution underlying each observed categorical variable [43]. In addition, standardised root mean residual (SRMR) was used, given that Shi and Maydeu-Olivares suggest this for estimating model fit, as other measures might be misleading when using DWLS [44]. Indication of a close fit was SRMR < 0.09, CFI > 0.95, TLI < 0.95, RMSEA < 0.05 and χ2/df < 3 [45].

To evaluate internal consistency, Cronbach’s alpha was calculated for each domain and for the scale as a whole. The internal consistency was considered good when Cronbach’s alpha was between 0.70 and 0.95 [42]. In all analyses, Jamovi version 2.2.5 was used. For the CFA, the SEM module in Jamovi was used.

## Results

### Characteristics of the study sample

In total, 89 participants responded to the MMD-HP. However, since respondents with more than ten per cent missing values were excluded from the analysis, the study sample dropped from 89 to 80. The study sample included women (n = 68) and men (n = 12), from various healthcare professions, such as enrolled nurses (n = 24), registered nurses (n = 34), physicians (n = 7), physio/occupational therapists (n = 15). They were at age 20–61 years, with work experience at the present workplace (median 6 years) and within the profession (median 14 years).

**Fig. 1:**
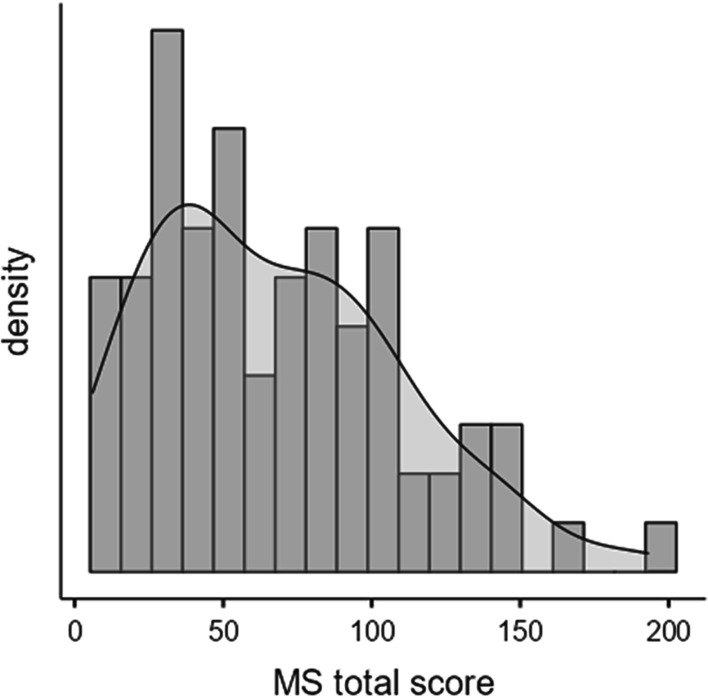
Distribution of MMD-HP total score

The MMD-HP total score ranged from 6 to 193, with a mean score of 67.81 (± 42.22). The distribution of missing values, that is, unanswered items, ranged from 3.4% to 9.0%. Items 21, 11 and 15 had the most significant missing values, with 7.9%, 9.0% and 9.0% missing values, respectively. An analysis showed ceiling effects (i.e. > 15%) on 13 items and floor effects on 21 items (Table 2). Figure 1 shows the distribution of the total score of the scale.

**Table 2:** Median and distribution in per cent for each item and response alternatives * (N = 89)

		Median	0 n (%)	1 n (%)	2 n (%)	3 n (%)	4 n (%)	Missing (%)
	*System-level*							
16	Be required to care for more patients than I can safely care for * Måste vårda fler patienter än jag kan ge säker vård till*	3.0	7 (7.9)	6 (6.7)	14 (15.7)	29 (32.6)	29 (32.6)	4 (4.5)
17	Experience compromised patient care due to lack of resources/equipment/bed capacity * Upplever att patientvården försämras på grund av bristande resurser/utrustning/ sängplatser*	3.0	12 (13.5)	6 (6.7)	18 (20.2)	30 (33.7)	19 (21.3)	4 (4.5)
19	Have excessive documentation requirements that compromise patient care * Upplever krav på omfattande dokumentation som medför försämrad patientvård*	2.0	20 (22.5)	19 (21.3)	29 (32.6)	10 (11.2)	5 (5.6)	6 (6.7)
23	Feel required to overemphasise tasks and productivity or quality measures at the expense of patient care * Känna krav att lägga tyngdpunkt på uppgifter gällande effektivitet eller kvalitetsskattningar på bekostnad av patientvården*	2.0	23 (25.8)	17 (19.1)	25 (28.1)	17 (19.1)	3 (3.4)	4 (4.5)
4	Be unable to provide optimal care due to pressures from administrators or insurers to reduce costs * Hindrad att ge optimal vård på grund av påtryckning från ledning för att minska kostnader*	2.0	25 (28.1)	8 (9.0)	11 (12.4)	20 (22.5)	22 (24.7)	3 (3.4)
7	Be required to care for patients whom I do not feel qualified to care for * Måste vårda en patient som jag upplever att jag inte är kompetent att vårda*	2.0	15 (16.9)	14 (15.7)	14 (15.7)	22 (24.7)	19 (21.3)	5 (5.6)
22	Be required to work with abusive patients/family members who are compromising quality of care * Måste arbeta med våldsamma patienter/närstående vilket riskerar att försämra vårdkvaliteten*	2.0	8 (9.0)	21 (23.6)	20 (22.5)	23 (25.8)	12 (13.5)	5 (5.6)
18	Experience lack of administrative action or support for a problem that is compromising patient care * Upplever brist på administrativt stöd för att hantera ett problem, med risk för försämrad patientvård*	1.0	19 (21.3)	23 (25.8)	23 (25.8)	13 (14.6)	5 (5.6)	6 (6.7)
	*Clinical causes*							
2	Follow the family’s insistence to continue aggressive treatment even though I believe it is not in the best interest of the patient * Följa närståendes begäran att fortsätta aggressiv behandling även om jag inte tror att det är för patientens bästa*	2.0	19 (21.3)	8 (9.0)	21 (23.6)	23 (25.8)	14 (15.7)	4 (4.5)
5	Continue to provide aggressive treatment for a person who is most likely to die regardless of this treatment when no one will make a decision to withdraw it * Fortsätta att ge aggressiv behandling till en patient som mest troligt kommer att dö oavsett denna behandling, när ingen vill ta beslut om att avsluta behandlingen*	3.0	14 (15.7)	10 (11.2)	14 (15.7)	26 (29.2)	21 (23.6)	4 (4.5)
1	Witness healthcare providers giving “false hope” to a patient or family * Bevittna vårdpersonal som ger”falskt hopp” till patient eller deras närstående*	2.0	21 (23.6)	15 (16.9)	23 (25.8)	22 (24.7)	4 (4.5)	4 (4.5)
3	Feel pressured to order or carry out orders for what I consider to be unnecessary or inappropriate tests and treatments * Känna press att ordinera eller utföra ordinationer gällande undersökningar och behandlingar som jag anser vara onödiga eller olämpliga*	2.0	13 (14.6)	17 (19.1)	33 (37.1)	18 (20.2)	5 (5.6)	3 (3.4)
8	Participate in care that causes unnecessary suffering or does not adequately relieve pain or symptoms * Delta i vård som orsakar onödigt lidande eller som inte tillräckligt lindrar smärta eller andra symtom*	2.5	15 (16.9)	11 (12.4)	16 (18.0)	22 (24.7)	20 (22.5)	5 (5.6)
10	Follow a physician’s or family member’s request not to discuss the patient’s prognosis with the patient/family * Följa läkarens eller närståendes begäran att inte diskutera patientens prognos med patienten/närstående*	1.0	29 (32.6)	14 (15.7)	18 (20.2)	16 (18.0)	6 (6.7)	6 (6.7)
	*Team/staff*							
21	Feel unsafe/bullied amongst my own colleagues * Känna mig otrygg/mobbad bland mina egna kollegor*	1.0	35 (39.3)	8 (9.0)	6 (6.7)	15 (16.9)	18 (20.2)	7 (7.9)
20	Fear retribution if I speak up * Rädsla för påföljder om jag påtalar brister*	1.0	29 (32.6)	24 (27.0)	11 (12.4)	8 (9.0)	11 (12.4)	6 (6.7)
11	Witness a violation of a standard of practice or a code of ethics and not feel sufficiently supported to report the violation * Bevittna överträdelser från vedertagna normer eller etiskt förhållningssätt och inte känna tillräckligt stöd för att rapportera det*	2.0	28 (31.5)	10 (11.2)	17 (19.1)	17 (19.1)	9 (10.1)	8 (9.0)
6	Be pressured to avoid taking action when I learn that a physician, nurse, or other team colleague has made a medical error and does not report it * Känna press att inte agera när jag får veta att en kollega eller annan teammedlem har gjort ett medicinskt misstag som inte rapporterats*	2.0	25 (28.1)	14 (15.7)	16 (18.0)	15 (16.9)	14 (15.7)	5 (5.6)
25	Work within power hierarchies in teams, units, and my institution that compromise patient care * Arbeta inom makthierarkier i teamet, på enheter eller på min avdelning vilket riskerar försämrad patientvård*	1.0	26 (29.2)	18 (20.2)	12 (13.5)	18 (20.2)	9 (10.1)	6 (6.7)
27	Work with team members who do not treat vulnerable or stigmatised patients with dignity and respect * Arbeta med teammedlemmar som inte bemöter sårbara eller stigmatiserade patienter med värdighet och respekt*	2.0	22 (24.7)	12 (13.5)	9 (10.1)	23 (25.8)	19 (21.3)	4 (4.5)
12	Participate in care that I do not agree with, but do so because of fears of litigation * Delta i vård som jag inte håller med om, men gör det på grund av rädsla för att bli anmäld*	1.0	37 (41.6)	5 (5.6)	17 (19.1)	12 (13.5)	12 (13.5)	6 (6.7)
	*Team/patient*							
15	Feel pressured to ignore situations in which patients have not been given adequate information to ensure informed consent * Känna press att ignorera situationer när patienten inte fått adekvat information för att kunna ge sitt samtycke*	2.0	23 (25.8)	16 (18.0)	20 (22.5)	18 (20.2)	4 (4.5)	8 (9.0)
14	Witness low quality of patient care due to poor team communication * Bevittna låg kvalitet på patientvården på grund av bristande kommunikation i teamet*	2.0	11 (12.4)	12 (13.5)	20 (22.5)	26 (29.2)	14 (15.7)	6 (6.7)
9	Watch patient care suffer because of a lack of provider continuity * Bevittna hur patientvården blir lidande på grund av dålig personalkontinuitet*	2.0	10 (11.2)	14 (15.7)	20 (22.5)	26 (29.2)	15 (16.9)	4 (4.5)
26	Participate on a team that gives inconsistent messages to a patient/family * Delta i ett team som ger motsägelsefull information till patient/närstående*	2.0	30 (33.7)	8 (9.0)	14 (15.7)	22 (24.7)	9 (10.1)	6 (6.7)
13	Be required to work with other healthcare team members who are not as competent as patient care requires * Förväntas arbeta tillsammans med teammedlemmar som inte har den kompetens som patientvården kräver*	3.0	18 (20.2)	9 (10.1)	13 (14.6)	25 (28.1)	19 (21.3)	5 (5.6)
24	Be required to care for patients who have unclear or inconsistent treatment plans or who lack goals of care * Måste vårda patienter med oklara eller inkonsekventa vård/ behandlingsplaner*	2.0	17 (19.1)	20 (22.5)	22 (24.7)	21 (23.6)	4 (4.5)	5 (5.6)

*The English original version in plain text, the Swedish version in italic

### Psychometric properties

The scale as a whole showed a Cronbach’s alpha of 0.96, with a range between 0.84 and 0.90 between the different subscales.

**Fig. 2:**
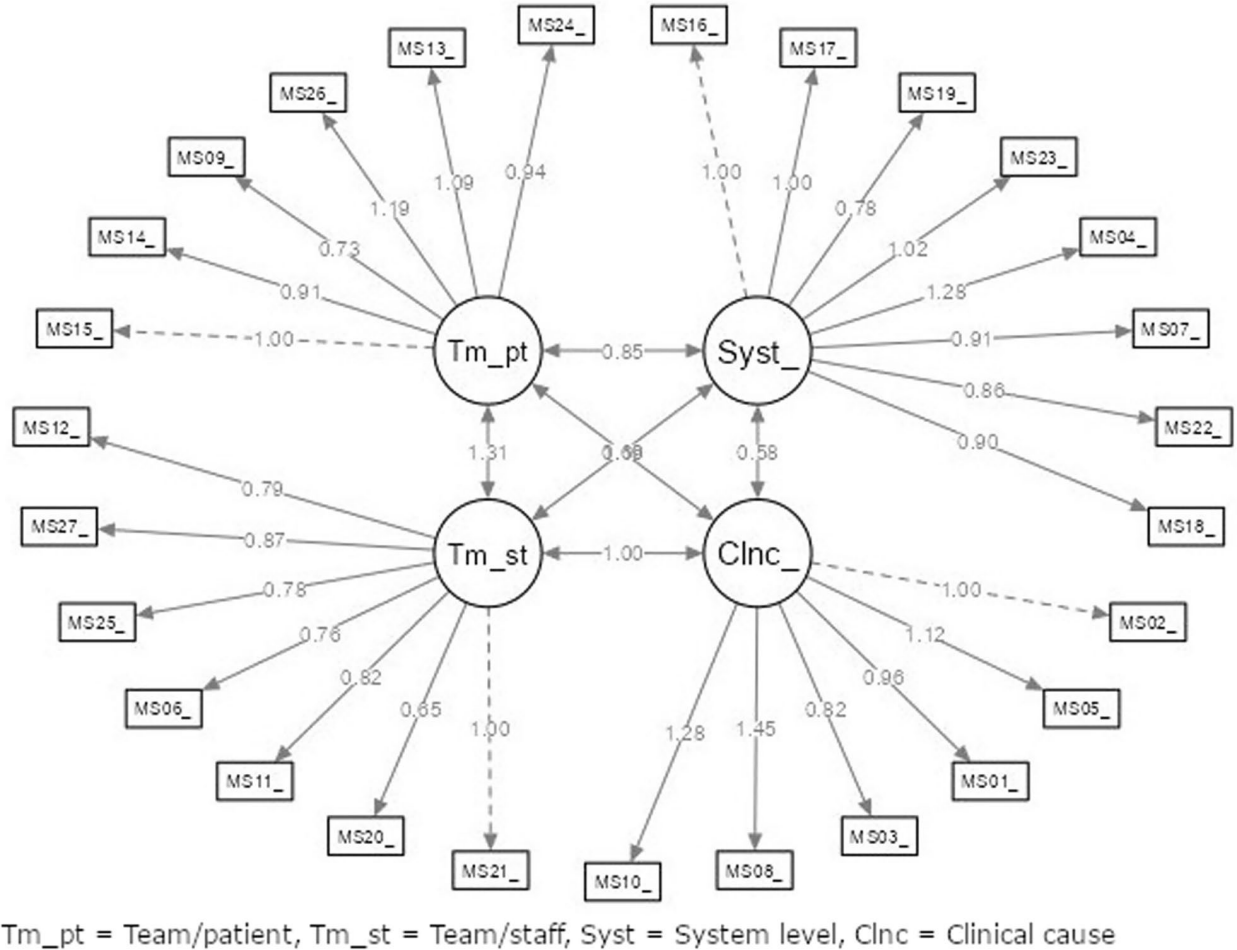
Model of the Swedish version of the MMD-HP-scale with factor loading

A confirmatory factor analysis based on the original four-factor structure showed good fit indices with a χ^2^ of 214.32 and a χ^2^/df of 0.67 (*p* = 1.000), CFI at 1.00, TLI at 1.02 and NFI at 0.97. RMSEA was at 0.00 and SRMR was at 0.08. Factor loadings for each domain are shown in Table 3 and Fig. 2. The results also highly correlated between the different domains (Table 4).

**Table 3:** Factor loadings and standardised factor loadings and Cronbach’s alpha of the Swedish version of MMD-HP

Domain	Item	Factor loading	Standardised factor loading	95% CI	*p*	*α*
System-level						0.84
Factor 1	16	1.00	0.00	1.00–1.00	< 0.01	
	17	1.00	0.07	0.87–1.14	< 0.01	
	19	0.78	0.06	0.66–0.90	< 0.01	
	23	1.02	0.08	1.12–1.45	< 0.01	
	4	1.28	0.08	1.12–1.45	< 0.01	
	7	0.91	0.07	0.77–1.04	< 0.01	
	22	0.86	0.06	0.74–0.99	< 0.01	
	18	0.90	0.06	0.78–1.02	< 0.01	
Clinical causes						0.87
Factor 2	2	1.00	0.00	1.00–1.00	< 0.01	
	5	1.12	0.09	0.95–1.30	< 0.01	
	1	0.96	0.08	0.80–1.11	< 0.01	
	3	0.82	0.07	0.70–0.95	< 0.01	
	8	1.45	0.10	1.24–1.65	< 0.01	
	10	1.28	0.09	1.10–1.46	< 0.01	
Team/Staff						0.90
Factor 3	21	1.00	0.00	1.00–1.00	< 0.01	
	20	0.65	0.04	0.57–0.72	< 0.01	
	11	0.82	0.04	0.74–0.91	< 0.01	
	6	0.76	0.04	0.67–0.84	< 0.01	
	25	0.78	0.04	0.70–0.87	< 0.01	
	27	0.87	0.05	0.78–0.96	< 0.01	
	12	0.79	0.04	0.70–0.88	< 0.01	
Team/patient						0.87
Factor 4	15	1.00	0.00	1.00–1.00	< 0.01	
	14	0.91	0.05	0.80–1.01	< 0.01	
	9	0.73	0.05	0.63–0.83	< 0.01	
	26	1.19	0.06	1.07–1.32	< 0.01	
	13	1.09	0.06	0.96–1.21	< 0.01	
	24	0.94	0.05	0.84–1.05	< 0.01

**Table 4:** Correlation between total score and the four different domains in the Swedish version of the MMD-HP-scale

	(1) MMD total score	(2) MMD System level	(3) MMD Clinical causes	(4) MMD Team/staff	(5) MMD Team/patient
1	–				
2	0.88***	–			
3	0.68***	0.40***	–		
4	0.82***	0.67***	0.45***	–	
5	0.92***	0.78***	0.53***	0.71***	–

***p < .001

## Discussion

This study indicates that the Swedish translation of MMD-HP is good and can be used to measure moral distress among health professionals in a Swedish context. The psychometric properties were found to be good to excellent. Grönlund and Brännström have previously reported the translation process of the instrument and found that it corresponded with the original version [39, 41]. Nevertheless, some of the items were adjusted to make them relevant in a Swedish context. However, this did not affect the Swedish translation’s psychometric properties.

We found that some of the items showed some skewness with a floor effect in most of the items. Further studies are necessary to address this. One reason for measuring this is to capture people or organisations experiencing high levels of moral distress [36]. Surprisingly, we found a ceiling effect in some of the items. However, we found that the instrument’s total score distribution broadly followed the same distribution as found by Fuji et al. [37]. However, data showed that the participants in our study rated moral distress lower compared to the Japanese nurses in that study.

All subscales, as well as the scale as a whole, showed good internal consistency with a Cronbach’s alpha of the different subscales of more than 0.84. Cronbach’s alpha for the instrument as a whole was 0.96. This is similar to the original version [36] of the MMD-HP where alpha was found to be 0.93, and a Japanese translation of the scale which showed an alpha for the total score of 0.91 [37]. This is in line with Tavakol and Dennik, who argue that alpha should range between 0.70 and 0.95 [46]. However, they also argue that an excessively high alpha value (> 0.90) may suggest redundancies [46].

Our confirmatory factor analyses showed that the Swedish translation of the MMD-HP had a good psychometric structure. The goodness of fit was acceptable, with an SRMR of 0.08. Cangur and Ercan argue that an SRMR value smaller than 0.10 indicates an acceptable fit, while an SRMR lower than 0.05 indicates a good fit [47]. One other reason to use SRMR as an indicator for goodness of fit is, according to Chen, that it is relatively independent of sample size [43]. It is, unfortunately, not possible to compare goodness of fit between our interpretation and the original instrument, since Epstein et al. did not perform a confirmatory factor analysis [36]. However, Fuji et al. found similar psychometric structure in their translation of the scale into Japanese, where they found CFI was 0.91, CFI/TLI 1.02 and RMSEA 0.061 [37], in line with our translation. Also, in the Spanish version, Girela-Lopez [48] found a goodness of fit with a CFI at 0.844 and RMSEA at 0.086.

In this study level of moral distress did not differ between groups concerning years of work experience. Rodriguez-Ruiz et al. used the Spanish version of MMD-HP and found that professionals who had fewer years of work experience, in an intensive-care context, experienced higher levels of moral distress [38].

As our result shows, we found support for the validity and reliability of the Swedish version of the MMD-HP. The use of MMD-HP may allow the possibility of assessing interventions such as ethics communication in groups among healthcare professionals by measuring their moral distress before and after experiencing ethically difficult situations. We argue that the MMD-HP may be an important cornerstone for understanding how healthcare professionals experience moral distress in their everyday clinical work. This knowledge may give insights among professionals concerning how to promote interventions in the workplace, especially when it comes to dealing with ethically difficult situations.

### Limitations

Our study has some limitations. We did not, as well as the original study, do any test–retest. This makes it difficult to fully know whether any difference in scoring is related to the impact of an intervention or whether the instrument is not stable over time. However, since moral stress cannot be expected to change very much over a short time, we believe that the instrument can still be used to evaluate the possible effects of an intervention performed over a preferable prolonged period. Nevertheless, further research still needs to be done to establish the stability and reliability of the instrument more rigorously. In addition, there is a risk that the model may be over-identified because CFI and RMSEA suggested a perfect fit. However, as we have seen in other models, we have found that when using DWLS as an estimator, these values tend to be very high (or low). The recommendation given by Shi and Maydeu-Olivares [44] is to use the standardised root mean residual (SRMR) since other measures might be misleading when using DWLS. Another weakness is that the instrument contains 27 items and can be time-consuming to complete, especially for people experiencing moral stress. Even though, as the original authors point out, the instrument can capture root causes of moral distress, further studies should be performed to see if any item reduction can shorten the instrument while still maintaining validity and reliability. Another weakness was that the sample in this study might appear somewhat limited as it was taken from an ongoing intervention for Clinical ethics support (CES) in which this instrument was used. This may have affected our results, but we believe the sample large enough to see if the Swedish translation corresponded to the English original. Furthermore, it would be of interest to examine whether there are some correlations between ethical climate and the different subscales.

## Conclusion

We found that the Swedish version of the MMD-HP has shown validity and reliability for use in a Swedish context for measuring moral distress among health personnel. In addition, the Swedish version is compatible with the original English version and can be compared with other similar studies. It is also helpful in measuring moral distress among healthcare workers but could be shortened to provide a faster and more pointed measure of moral distress in a workplace.

## Data Availability

The datasets used and/or analysed during the current study are available from the corresponding author on reasonable request.

## Abbreviations

MMD-HP: Measure of moral distress for healthcare professionals
MDS-R: Moral distress scale-revised
CFI: Comparative fit index
TLI: Tucker-Lewis index
RMSEA: Root mean square error of approximation
SRMR: Standardised root mean squared residual
DWLS: Diagonally weighted least squares
